# Evaluation of EMG patterns in children during assisted walking in the exoskeleton

**DOI:** 10.3389/fnins.2024.1461323

**Published:** 2024-10-24

**Authors:** Margherita Villani, Priscilla Avaltroni, Giulia Scordo, Damiana Rubeca, Peter Kreynin, Ekaterina Bereziy, Denise Berger, Germana Cappellini, Francesca Sylos-Labini, Francesco Lacquaniti, Yury Ivanenko

**Affiliations:** ^1^Laboratory of Neuromotor Physiology, Istituto di Ricovero e Cura a Carattere Scientifico Fondazione Santa Lucia, Rome, Italy; ^2^Department of Systems Medicine and Center of Space Biomedicine, University of Rome Tor Vergata, Rome, Italy; ^3^ExoAtlet, Esch-sur-Alzette, Luxembourg

**Keywords:** children, robotic exoskeleton, assisted gait, EMG patterns, spinal locomotor output, development

## Abstract

While exoskeleton technology is becoming more and more common for gait rehabilitation in children with neurological disorders, evaluation of gait performance still faces challenges and concerns. The reasoning behind evaluating the spinal locomotor output is that, while exoskeleton's guidance forces create the desired walking kinematics, they also affect sensorimotor interactions, which may lead to an abnormal spatiotemporal integration of activity in particular spinal segments and the risk of abnormalities in gait recovery. Therefore, traditional indicators based on kinematic or kinetic characteristics for optimizing exoskeleton controllers for gait rehabilitation may be supplemented by performance measures associated with the neural control mechanisms. The purpose of this study on a sample of children was to determine the basic features of lower limb muscle activity and to implement a method for assessing the neuromechanics of spinal locomotor output during exoskeleton-assisted gait. To this end, we assessed the effects of a robotic exoskeleton (ExoAtlet Bambini) on gait performance, by recording electromyographic activity of leg muscles and analyzing the corresponding spinal motor pool output. A slower walking setting (about 0.2 m/s) was chosen on the exoskeleton. The results showed that, even with slower walking, the level of muscle activation was roughly comparable during exoskeleton-assisted gait and normal walking. This suggests that, despite full assistance for leg movements, the child's locomotor controllers can interpret step-related afferent information promoting essential activity in leg muscles. This is most likely explained by the active nature of stepping in the exoskeleton (the child was not fully relaxed, experienced full foot loading and needed to maintain the upper trunk posture). In terms of the general muscle activity patterns, we identified notable variations for the proximal leg muscles, coactivation of the lumbar and sacral motor pools, and weak propulsion from the distal extensors at push-off. These changes led to the lack of characteristic lumbosacral oscillations of the center of motoneuron activity, normally associated with the pendulum mechanism of bipedal walking. This work shows promise as a useful technique for analyzing exoskeleton performance to help children develop their natural gait pattern and to guide system optimization in the future for inclusion into clinical care.

## Introduction

An important therapeutic goal for children with developmental disorders is helping them learn to walk independently and efficiently (Willoughby et al., 2009; Smania et al., 2011; Anderson et al., 2013; Graham et al., 2016; Cappellini et al., 2020). This is also essential to their social and psychological growth since development, social engagement, and social status can all suffer from immobility (Lancioni et al., 2009; Whittingham et al., 2010; Kerr et al., 2011; Anderson et al., 2013). The development of tools and biotechnology advancements can greatly aid children optimize their physical potential, retain their functional skills throughout adulthood, and improve their walking.

The use of wearable exoskeletons and gait orthoses is one of the most innovative approaches to gait support and rehabilitation, as studies have shown that repeated locomotor training improves children's gait characteristics and walking abilities (Kuenzle and Brunner, 2009; Smania et al., 2011; Lerner et al., 2017; Karunakaran et al., 2020; Zistatsis et al., 2021; Hunt et al., 2022; Kuroda et al., 2023; McCormick et al., 2023). Robotic gait training represents a new frontier in the world of pediatric rehabilitation and early locomotor function stimulation can also improve the course of motor development by allowing children to participate in motor learning experiences, which include playful self-discovery and exploration (Cappellini et al., 2020). Recent advances in exoskeleton technology have resulted in the development of multifunctional exoskeletons for children, such as the ExoAtlet exoskeleton, of which there are pediatric and adult variants (see https://exoatlet.lu/bambini/; Shapkova et al., 2020; Baptista et al., 2022). For clinical use, the ExoAtlet Bambini exoskeleton combines walking and standing aid and is designed for children with severe gait impairments to ambulate with hands-free support, assisting various locomotor tasks and increasing the capacity for activity and participation. The current study looks at the characteristics of walking in this exoskeleton in youngsters.

When applying this technology for gait assistance and rehabilitation, it is also critical to consider human-machine interactions and the user's adaptation to the exoskeleton (Massardi et al., 2022). Different exoskeletons (powered, passive, with varying degrees of freedom, etc.) are being developed and proper protocols and metrics for their assessment can help with the control implementations of these exoskeletons (Pinto-Fernandez et al., 2020; Remazeilles et al., 2022). Assessments based on kinematic/kinetic data prevail for evaluation of walking in exoskeletons although conclusions regarding neural control mechanisms and sensorimotor adaptations are limited. Furthermore, certain kinematic parameters are a consequence of the control implemented on the exoskeleton. Therefore, traditional indicators based on kinematic or kinetic variables could be supplemented by performance measures related to the neural control strategy (Zhvansky et al., 2022). Indeed, neurologically intact individuals walking in an exoskeleton may show notable differences in muscle coordination patterns (Sylos-Labini et al., 2014; Steele et al., 2017; Rinaldi et al., 2020). A better match between the subject's locomotor output and the control strategy of a certain exoskeleton could likely be a goal for optimizing powered exoskeletons in order to achieve normal functioning of the spinal locomotor pattern generation circuitry. In addition, understanding what occurs at the spinal cord output level may be essential for gait rehabilitation, as problems in the recovery of gait may arise from abnormal spatiotemporal integration of activity in particular spinal segments (Ivanenko et al., 2009; Martino et al., 2018; Cappellini et al., 2020).

This study's objectives were to identify the basic features of lower limb muscle activity and implement an evaluation technique for the neuromechanics of spinal locomotor output during exoskeleton-assisted locomotion on a sample of children. In order to better understand the performance of the ExoAtlet Bambini exoskeleton in terms of spinal motor output characteristics, we have reported here these characteristics in volunteers who were healthy youngsters. While the ExoAtlet exoskeleton is designed for children aged 2–15 yrs and can be tailored to the child's anthropometric parameters, we used its version for older children (ExoAtlet Bambini MIDI) and focused this study on a sample of participants who were 7–11 years old. We were able to assess its performance in children of a comparable age and also to rule out the potentially confounding influence of age since the spinal locomotor output undergoes functional maturation, especially in younger children (Ivanenko et al., 2013). Walking in the exoskeleton and normal walking at a self-selected speed were compared. In particular, the assessment technique involved a comparison of activity of flexor and extensor muscle groups of the lower limbs and mapping the recorded patterns of muscle activity onto the approximate rostrocaudal location of the motoneuron (MN) pools in the spinal cord. To this end, we completed simultaneous kinematic and electromyographic (EMG) recordings in several muscles, which provide an indirect measure of the net firing of MNs of those muscles in the corresponding spinal cord segments (Yakovenko et al., 2002; Grasso et al., 2004; Ivanenko et al., 2006; Monaco et al., 2010; Wenger et al., 2016).

## Materials and methods

### Participants

Experiments were performed in the Laboratory of Neuromotor Physiology of IRCCS Santa Lucia Foundation in conformity with the Declaration of Helsinki for research with human subjects, and following the procedures of the Ethics Committee of the Santa Lucia Foundation for walking in the exoskeleton. In particular, we were allowed to study only six healthy children with no known neurological disorders or other impairments that would have prevented them from walking in the exoskeleton, since the study did not bring therapeutical benefits. Their characteristics are listed in Table 1. The parents of each child were informed about the study purpose, duration and structure, and provided informed written consent.

**Table 1:** Participant characteristics and general gait parameters (mean ± SD).

**Participants**	**Age (yrs)**	**Gender**	**Height (m)**	**Weight (kg)**	**NW**				**EXO**			
					**Mean speed (m/s)**	**# strides**	**Stride length (L)**	**Stride duration (s)**	**Mean speed (m/s)**	**# strides**	**Stride length (L)**	**Stride duration (s)**
S1	7	M	1.25	24	0.88 ± 0.13	5	1.66 ± 0.10	1.13 ± 0.07	0.19 ± 0.03	11	0.84 ± 0.13	2.98 ± 0.51
S2	8	M	1.32	29	0.78 ± 0.05	6	1.31 ± 0.06	1.16 ± 0.04	0.17 ± 0.01	6	0.69 ± 0.08	2.61 ± 0.06
S3	8	M	1.36	29	0.57 ± 0.33	27	1.18 ± 0.37	1.56 ± 0.51	0.18 ± 0.07	12	0.94 ± 0.09	4.06 ± 0.62
S4	9	M	1.30	29	0.67 ± 0.04	8	1.47 ± 0.06	1.35 ± 0.08	0.19 ± 0.03	17	0.81 ± 0.09	3.05 ± 0.51
S5	10	F	1.43	32	0.81 ± 0.19	5	1.40 ± 0.28	1.40 ± 0.24	0.27 ± 0.09	6	0.86 ± 0.05	2.63 ± 0.03
S6	11	F	1.46	33	0.75 ± 0.35	16	1.28 ± 0.26	1.56 ± 0.46	0.19 ± 0.02	9	0.90 ± 0.08	4.06 ± 0.12

Stride length was normalized to the limb length L.

### Brief description of the *ExoAtlet* bambini exoskeleton

There is a limited number of devices currently available for the pediatric use. Some are significantly different in use mode, such as Lokomat (De Luca et al., 2022) or Gait Trainer (Smania et al., 2011), which are in fact stationary devices utilizing treadmill for walking, while other robotic devices for overground walking provide only local joint assistance or have restrictions for balance and motion (e.g., Bayón et al., 2018; Zistatsis et al., 2021; Hunt et al., 2022; Kuroda et al., 2023). The closest analog for children would be the Angel Legs M20 providing assistance torques at hip and ankle joints according to the gait cycle and to the patient's residual muscle strength (although for relatively older children as currently stated) (Choi et al., 2024) and ATLAS2030 operating in a similar way, but not allowing the accompanying personnel to control the weight transfer of the pilot, utilizing a safety frame (Delgado et al., 2021; Castro et al., 2024). Compared to tethered robots, overground exoskeletons appear to be more effective for dynamic balancing, allowing proper body alignment during weight-shifting actions. Effective neurorehabilitation requires active participation in the training process. Children that receive overground gait training are able to explore and navigate in a more natural setting, engage their neuromuscular system actively, and improve their stride-to-stride variability.

The ExoAtlet Bambini MIDI wearable exoskeleton is designed to enable children with lower limb disabilities to walk, supporting assisted walking and locomotor training under gravitational load (https://exoatlet.lu/bambini/, EC certificate n.0068/QPZ-DM/206-2020). This aid promotes the development of functional residual resources necessary for the walking cycle in children with neuromotor disabilities. The ExoAtlet Bambini is intended for treatment of the full scope of walking disorders of children with any level of remaining motion in lower limbs. It has six actuators (hip, knee and ankle on each leg moving in sagittal plane) and two optional ones (for the hip rotation in the frontal plane) and is normally assisted by a therapist controlling the balance and weight transfer of the patient. Optionally, upon the approval of the therapist, a safety frame can be used to ensure the stability of the patient walking alone.

**Figure 1:**
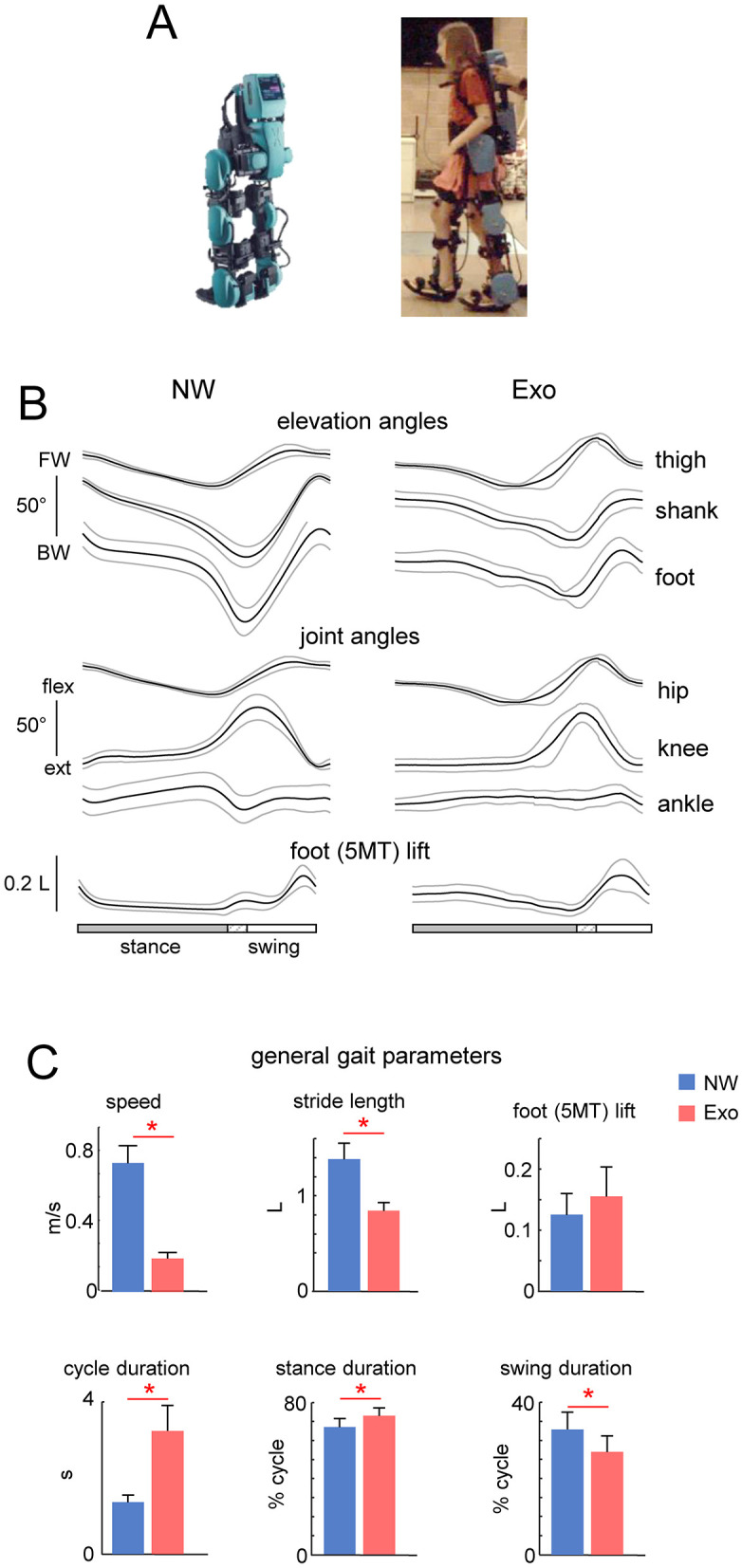
General characteristics of normal and exoskeleton-assisted walking. **(A)** View of the exoskeleton. **(B)** Ensemble-averaged (mean ± SD across children) foot, thigh and shank elevation angles (FW, forward direction and BW, backward direction), and hip, knee and ankle joint angles plotted vs. normalized gait cycle. **(C)** General gait parameters (walking speed, stride length, foot (5MT, fifth metatarso-phalangeal joint of the foot) lift, cycle duration, and relative stance and swing durations). Stride length and foot lift were normalized to the limb length L (thigh + shank). Asterisks denote significant differences (RM ANOVA with Tukey's HSD, *p* < 0.05) between conditions.

The exoskeleton weighs 17 kg, including the battery, and supports itself by transferring weight to the ground via its footplates. It is secured to the wearer at five major points: the footplate, shank, thigh, pelvis, and torso (Figure 1A). Its shank, thigh and foot segment lengths, as well as pelvic widths, can be accommodated to meet varying subject heights and sizes (weight up to 60 kg, height 1.2–1.6 m). Footplates composed of carbon fiber are designed to accommodate human feet, with one degree of freedom (ankle dorsi/plantar flexion) passively sprung with a certain stiffness. Specifically, the exoskeleton was adjusted in two ways. First, the user's anthropometric parameters were matched mechanically: shank and thigh lengths were chosen to match the rotation axes of the exoskeleton with the physical features of the user. Furthermore, the width of the exoskeleton was adjusted to ensure tight and comfortable placement of the user inside the device. Additionally, the construction of the exoskeleton allows adjustment of all leg fasteners in sagittal and frontal planes to match the anthropometric characteristics of the user. The exoskeleton range of motion is capable of supporting all operating modes: from sitting with the knees and hips bent at 90 degrees to standing with 0 degrees extension. Therefore, the second stage of setting up the exoskeleton included setting the specific gait parameters making the exercise comfortable for the user. After donning the exoskeleton, the child was moved from a sitting position to a standing position and vice versa using the exoskeleton's sit-to-stand and sit-down control modes, respectively. The exoskeleton itself is capable of walking at various speeds up to 0.33 m/s and with the step height/length up to 15/30 cm. The exoskeleton is made to be easily put on and taken off: a trained therapist can do it in < 3 min.

The exoskeleton is operated by a PC tablet embedded into its back or, if the user chooses to operate it independently, by detaching the tablet from the exoskeleton and holding it in their own hand. The exoskeleton comprises six motors (hip, knee, and ankle on each leg) allowing enough degrees of freedom to simulate the natural movement of the lower limb during the gait. All motors are independently controlled allowing adjustments of the gait parameters as needed. During this study, the ankle, although motorized, was not involved in the movement to full possible extent. This may be suitable for children with limited ankle flexibility, but it may require updates to include full rolling gait phase. Such limitation in ankle movement is now determined by the gait pattern of the exoskeleton. Currently, it is defined by the limited motion ability of the ankle joint of the majority of users and how they use the orthosis. Further research is necessary to determine the most clinically useful update of this feature to ensure proper foot rolling phase of the gait, but also maintaining the ability to use the device for the patients with severely limited ankle range of motion. Adjustable walking parameters include step length, height and speed, and the time delay between steps. The control modes allow standing still, stepping in place, standing up and sitting down on a chair, level walking with different cycle durations and in combination with functional electrical stimulation of leg muscles, walking on inclined surface, backward walking, stepping over obstacles, and comfortable walking up and down stairs. If excessive torque is sensed, sensors embedded into each motor will automatically stop stepping (Berezij et al., 2017). In this study, we used the default settings for forward level walking at ~0.2 m/s and without a time delay between steps (the latter mode is intended for patients who are either at the beginning of their exoskeleton training regimen or have severe gait and posture abnormalities and need to move more slowly). The kinematic and gait parameters obtained are plotted in Figure 1. The exoskeleton was designed to walk either with crutches (since it cannot provide full balance), or with assistance of the therapist. In the latter situation (used in this study), the design features two rear handholds for one or two assistants to provide physical assistance or safety. A view of the exoskeleton in depicted in Figure 1A.

### Protocol

Two experimental conditions were recorded in the same experimental session: normal walking at self-selected speed without the exoskeleton (NW), and exoskeleton-assisted walking (Exo). The participants were asked to walk along a 6–8-m walkway while kinematics and leg muscle EMG activity were recorded. We started with recording walking in the exoskeleton and then normal walking. In the first condition, the exoskeleton was first fitted to the subject in the sitting position. The EMG electrodes for recording muscle activity (see below) were placed on the leg's skin in the same position during Exo and NW trials. The subjects were instructed to remain passive and not to walk voluntarily while the exoskeleton was activated, they were only asked to pay attention to the arms to avoid making contact with the exoskeleton in the vicinity of the pelvis and hip joints, which never happened. Nevertheless, as is the case with adults passively moving by the exoskeleton (Sylos-Labini et al., 2014), adaptation and muscle activity were expected to be induced (see Results). To begin and terminate the Exo experiments, the subjects were made to stand using the get up and sit-down control modes of the exoskeleton. For security reasons, the experimenter was always behind the child, giving hand contact via rear handholds. This interaction did not influence gait and was simply intended to ensure balance and prevent a potential fall, which never occurred. Because children did not use crutches for stability, they were free to move their upper limbs. While the ExoAtlet Bambini exoskeleton supports a variety of control modes, in this study we specifically focused on “default” level walking at a slow speed, which is usual for secure walking in the exoskeleton. Slow overground walking using a powered exoskeleton is also common in clinical settings for gait assistance and rehabilitation in children (Karunakaran et al., 2020; Chen et al., 2021; Kuroda et al., 2023). It allowed us to evaluate and compare the neuromechanics of the spinal locomotor output during walking in the exoskeleton and during normal walking. Typically, we collected the data from 2 to 6 trials while walking in the exoskeleton following a short period of training (1–2 trials), in which children simply needed to familiarize with the guidance of the exoskeleton. On average, 6–11 strides were recorded and analyzed in each subject in each experimental condition (Table 1). Gait initiation and gait termination steps were excluded from the analysis. The total duration of the experimental session was about 1 h.

### Data recording

Bilateral full-body kinematics was recorded at 200 Hz by means of the Vicon-Nexus system (Oxford, UK) with 10 cameras placed around the walking path. Infrared reflective markers were attached on each side of the child to the skin overlying the following landmarks: greater trochanter (GT), lateral femur epicondyle (KNEE), ankle (ANK) and the fifth metatarso-phalangeal joint of the foot (5MT). In the case of Exo walking, these markers were attached on the exoskeleton in correspondence to appropriate landmarks. Electromyographic (EMG) activity was recorded by means of surface electrodes. The following eight muscles of the right leg were analyzed: rectus femoris (RF), vastus lateralis (VL), vastus medialis (VM), biceps femoris (long head) (BF), semitendinosus (ST), gastrocnemius lateralis (LG), soleus (SOL), and tibialis anterior (TA). We have previously demonstrated that the major loci of motoneuron activity are similar and can be identified using a set of recorded muscles representing major extensor and flexor groups (Ivanenko et al., 2013; La Scaleia et al., 2014). All EMGs were recorded at 2,000 Hz using the wireless Trigno EMG system (Delsys Inc., Boston, MA), bandwidth of 20–450 Hz, overall gain of 1,000. Sampling of kinematic and EMG data were synchronized.

### Data analysis

#### Kinematics

The lower limb was modeled as an interconnected chain of rigid segments: GT–KNEE for the thigh, KNEE-ANK for the shank, and ANK– 5MT for the foot. Gait cycle was defined as the time between two successive foot–floor touchdown contacts by the same leg (Dominici et al., 2007) according to the local minima of the vertical displacement of the ANK marker. The timing of the lift-off was determined similarly (when the 5MT marker was elevated by more than 2 cm). Walking speed for each stride for overground walking with (“Exo”) and without (“NW”) the exoskeleton was computed as the mean speed of the horizontal trunk movement, the latter being identified by the time course of the displacement of the virtual marker located at the midpoint between left and right GT markers. Stride length was measured according to horizontal displacement of the foot (5MT) maker. The stride length and foot trajectory were normalized to the limb length (L, determined by summing lengths of the thigh and shank segments) of the participants. Data were time-interpolated over individual gait cycles to fit a normalized 200-point time base.

#### EMG patterns

The raw EMG signals were high-pass filtered (60 Hz), full-wave rectified and low-pass filtered with a zero-lag fourth-order Butterworth filter (5 Hz) to obtain envelope time series (Sylos-Labini et al., 2014, 2020). Similarly, to the kinematic data, the processed EMG data were time-interpolated over a normalized 200-point time base *t*, and were averaged across all cycles.

To characterize differences in the timing of EMGs, we computed the center of muscle activity (*CoMA*) (Cappellini et al., 2016). This parameter was calculated over individual strides and then averaged across cycles. The *CoMA* was calculated using circular statistics (from Equations 1–3) as the angle of the vector (1st trigonometric moment) in polar coordinates (polar direction denoted the phase of the gait cycle, with angle θ that varies from 0 to 360°) that points to the center of mass of that circular distribution of the *i*_*th*_ muscle EMG using the following equations:


(1) $$\begin{array}{c} A=\overset{200}{\underset{t=1}{\sum }}\left(\cos\theta \left(t\right)\times EMG_{i}\left(t\right)\right) \end{array}$$

(2) $$\begin{array}{c} B=\overset{200}{\underset{t=1}{\sum }}\left(\sin\theta \left(t\right)\times EMG_{i}\left(t\right)\right) \end{array}$$

(3) $$\begin{array}{c} CoMA=\tan^{-1}\left(\frac{B}{A}\right) \end{array}$$

where *t* = 1:200 is a normalized time base. The *CoMA* provides only an estimate of the timing of the EMG bursts and was chosen because it was impractical to reliably identify a single peak of activity in some muscles (e.g., TA).

#### Spinal maps

To characterize the spatiotemporal organization of the total motor output and to reconstruct the maps of spinal motoneuron (MNs) activity, the EMG envelops were mapped onto the approximal rostrocaudal location of MNs-pools in the human spinal cord derived from published literature, providing an interpretation of the motor pool activation at a segmental level rather than at the individual muscle level (Yakovenko et al., 2002; Ivanenko et al., 2006, 2013). We used the myotomal maps of Kendall et al. (2005) to determine the approximate rostro-caudal location of MNs pools in the human spinal cord; the rectified EMG provides an indirect measure of the net firing of MNs of that muscle. We used the non-normalized procedure (EMGs were expressed in microvolts, μV, Ivanenko et al., 2008) to characterize MN activity in the corresponding spinal segment *S*_*J*_:


(4) $$\begin{array}{c} S_{j}\left(t\right)=\;\frac{\sum_{i=1}^{n_{j}}\;k_{ij}\;.\;EMG_{i}\;\left(t\right)}{n_{j}} \end{array}$$

where *n*_*j*_ is the number of *EMG*_*i*_ waveforms corresponding to the *j*_*th*_ segment and *k*_*ij*_ is the weighting coefficient for the *i*_*th*_ muscle (*X* and *x* in Kendall's chart were weighted with *k*_*ij*_=1 and *k*_*ij*_=0.5, respectively). To visualize a continuous smoothed rostrocaudal spatiotemporal activation of the spinal cord, we used a filled contour plot that computes isolines calculated from the activation waveform matrix and fills the areas between the isolines using separate colors. We calculated the center of activity (CoA) of the six (from L2 to S2) most active lumbosacral segments using the following equation:


(5) $$\begin{array}{c} CoA(t)=\;\frac{\sum_{j=1}^{N}\;S_{j}(t)\;\times j}{\sum_{j=1}^{N}\;S_{j}(t)\;} \end{array}$$

where *S*_*j*_ is the estimated activity (from Equation 4) of the *j*_*th*_ segment (the origin for the CoA and for the vector *j* =[1 2 3 4 5 6] being defined as the caudal-most segment), and *N* = 6 is the number of spinal segments. Based on this technique, we reconstructed the total unilateral motor output of the lumbosacral enlargement and these maps were compared between the two conditions we studied (Exo vs. NW). In particular, we calculated the timing of the maximal activation of upper lumbar (L3 + L4) and sacral (S1 + S2) segments throughout the gait cycle (Ivanenko et al., 2013). In addition, we computed the correlation coefficients between the CoA (from Equation 5) of single subjects during Exo and NW with the averaged (across subjects) CoA during NW. Similarly, we computed the similarity (correlation) of sacral or lumbar motor pool activation in the Exo condition with the averaged (across subjects) sacral or lumbar activation during NW.

Finally, since Exo walking in children was slower (about 0.2 m/s, see Results) than NW, we verified whether the observed differences between Exo and NW could be accounted for by the effect of speed. Even though we did not record Exo walking at higher speeds, we evaluated the effect of speed on the spatiotemporal characteristics of motor pool output during normal walking. During NW, individual strides ranged in walking speed from 0.14 to 1.3 m/s, and we divided the strides into three ranges to investigate the potential influence of speed: 0.14–0.42 m/s, 0.42–0.83 m/s, and 0.83–1.3 m/s.

### Statistics

Descriptive statistics included the calculation of the mean and standard deviation (SD) of the assessed variables. The experimental data set met the normal distribution criteria (the Shapiro–Wilk W-test, *p* > 0.05), therefore parametric statistics were used for statistical data analysis. One-way ANOVA was used to evaluate the effect of group on different variables (speed, stride length, foot lift, cycle duration, stance duration, swing duration, and spinal output correlations). If ANOVA resulted in a significant effect, then a Tukey HSD (Honestly Significant Difference) *post-hoc* test was used to detect differences between groups. Statistics on correlation coefficients for spinal maps analysis was performed on the normally distributed, *Z*-transformed values. Statistical analysis of circular data (Batschelet, 1981) was used to characterize the mean orientation of the CoMA, its variability across strides and the max timing of the lumbar and sacral segments' activation. The Watson-Williams test was used for the *post-hoc* comparison. Reported results are considered significant for *p* < 0.05.

## Results

### General performance

Figure 1 (upper panels) illustrates the ExoAtlet exoskeleton. Given the design of the exoskeleton, which offered stability through trunk support and two rear handholds for an experimenter for safety, children never fell and were also free to move their arms. All children succeeded to complete the protocol, walking in the exoskeleton was relatively slow and general gait parameters are shown in Figure 1. Figure 1B illustrates ensemble-averaged angular movements in the two walking conditions (NW and Exo). The shank and foot elevation angles had a greater range of motion (ROM) during NW than during Exo, which was related to a higher walking speed and a larger stride length. Despite differences in walking speed, hip and knee flexion angles were similar because, in the Exo control implementation, they were slightly increased during swing to ensure sufficient foot clearance (see a bell-shaped foot trajectory with a maximum around midswing, Figure 1A bottom) and avoid potential stumbling, which never occurred.

Figure 1C illustrates general gait parameters. NW was faster than Exo walking (0.77 ± 0.10 m/s vs. 0.2 ± 0.03 m/s, respectively, *p* < 0.01 Tukey HSD). This resulted in a significant difference in the mean cycle duration (1.4 ± 0.2 s vs. 3.2 ± 0.7 s, *p* < 0.01) with a shorter relative stance duration (67.1 ± 4.5% vs. 73.0 ± 4.2% of cycle, *p* < 0.05) and a longer relative swing duration (32.9 ± 4.5% of cycle vs. 27.0 ± 4.2% of cycle, *p* < 0.05) for the NW condition. There was also a significant difference in stride length (normalized to limb length, 1.39 ± 0.17 vs. 0.85 ± 0.09, *p* < 0.01). There were no significant differences in foot lift (Figure 1C).

### Evaluation of EMG patterns

**Figure 2:**
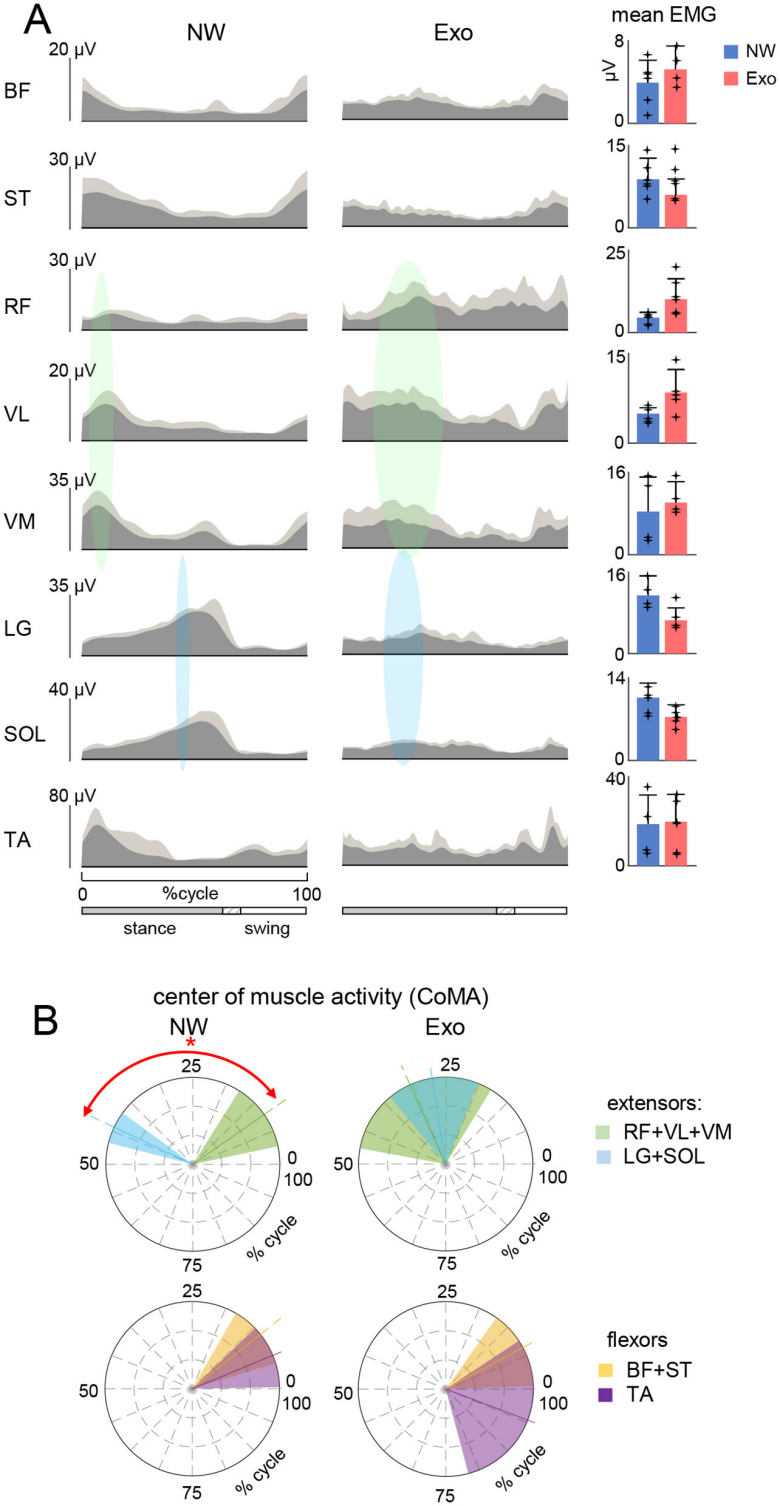
Characteristics of EMG activity. **(A)** Ensemble averaged (in dark gray, +SD in light gray) EMG activity patterns of 8 muscles of the right leg recorded in the two conditions (NW and Exo). EMG data are plotted vs. normalized gait cycle. As the relative duration of stance varied, a hatched region indicates an amount of variability (range) in the stance phase duration across participants. Mean EMG activities (+SD, and each point represents the data for an individual child) of the corresponding muscles are shown on the right. EMG waveforms of each muscle were averaged across strides of each participants, and then averaged across participants. Mean EMGs (right panel) were calculated similarly. The timing of the primary action of the proximal (in light green) and distal (in sky-blue) extensors is represented schematically by colored ellipses: the timing and the width of ellipses correspond to the CoMA and its angular SD plotted in panel **(B)**. **(B)** Polar plots of the center of muscle activity (CoMA) of the major groups of muscles. Even though some muscles are biarticular and act on different joints, we identified them as proximal (RF + VL + VM) and distal (LG + SOL) extensors, as well as proximal (BF + ST) and distal (TA) flexors. Polar direction denotes the relative time of the CoMA over the gait cycle (time progresses counter-clockwise) and the width of the sector denotes angular SD. Asterisk denotes significant difference (circular Watson-Williams test, *p* < 0.05).

Figure 2A shows ensemble-averaged EMG patterns (left panels) and the mean levels of activity (right panels) in NW and Exo conditions. Some muscles (e.g., LG, SOL) tended to decrease their activity during Exo walking, while others (e.g., VL, VM, RF) tended to increase it, albeit not significantly. In particular, Exo walking showed substantial activity in the ankle extensors (LG, SOL) throughout the stance phase, but there was no noticeable burst of activity in late stance related with propulsion during NW. The activity of proximal extensors (RF, VL, VM) typically occurred at early stance (~10% of gait cycle) during weight acceptance in NW, whereas in the Exo condition it often occurred in midstance as well (~30% of gait cycle, highlighted schematically by color regions in Figure 2A).

Although some muscles act on different joints, we classified their primary function as proximal (RF + VL + VM) and distal (LG + SOL) extensors, as well as proximal (BF + ST) and distal (TA) flexors, and plotted the corresponding center of activity CoMA in Figure 2B. There was a clear differentiation of the CoMA between proximal (RF + VL + VM) and distal (LG + SOL) extensors only during NW (*p* < 0.01, Watson-Williams test). The same differentiation was not present in proximal (BF + ST) and distal (TA) flexors in both conditions. Nevertheless, regardless of above-mentioned variations in the EMG envelopes and interindividual variability, it is worth noting that, despite a slower speed and fully assisted gait in the Exo condition, the overall level of EMG activity was comparable to that during NW (Figure 2A, right panel).

### Spinal maps of MN activity

**Figure 3:**
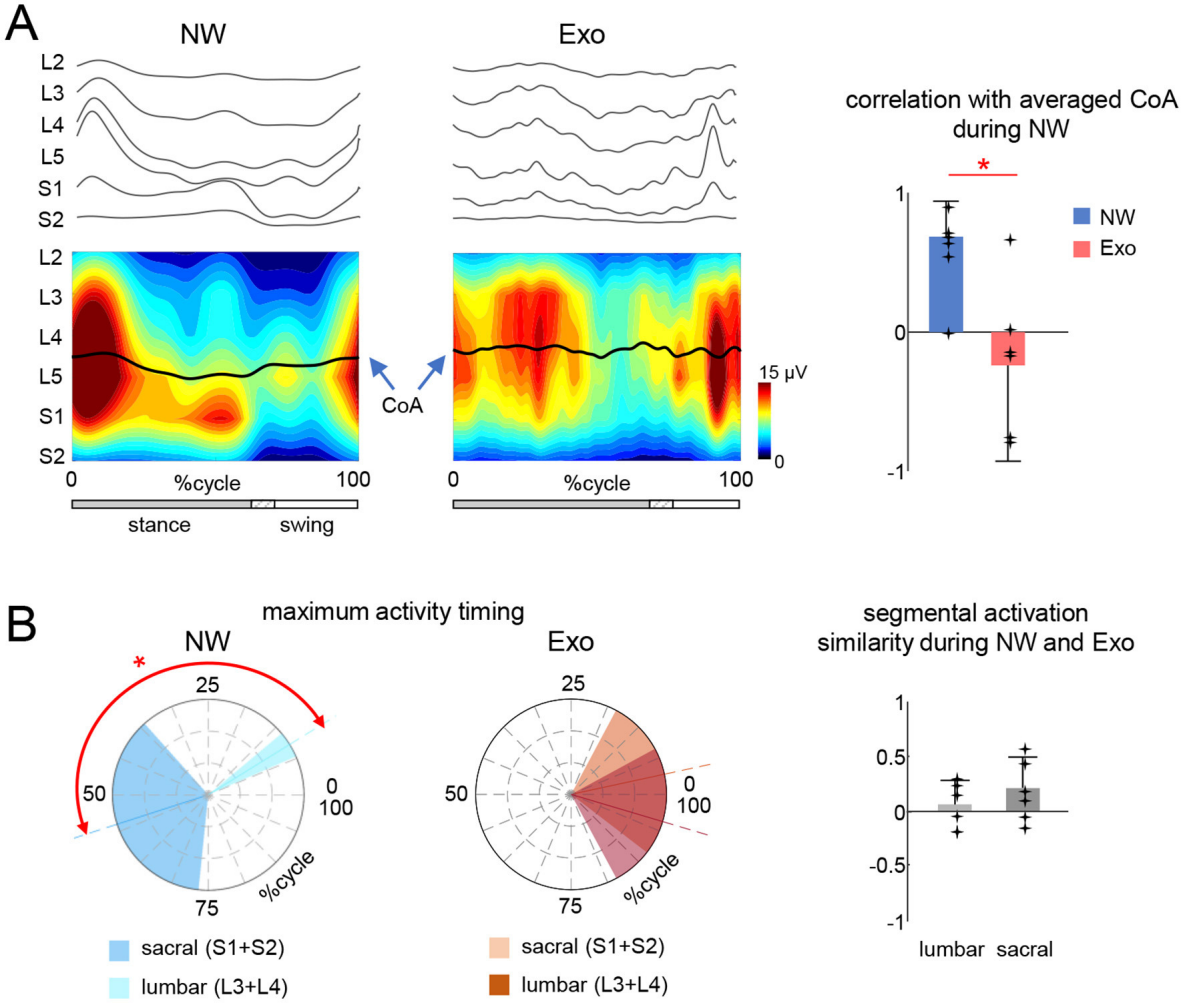
Spinal locomotor output in children during normal and exoskeleton-assisted walking. **(A)** Spatiotemporal maps of motoneuron activity of the lumbosacral enlargement reconstructed using ensample-averaged EMG envelopes. Output pattern of each segment is shown in the top panels while the same pattern is plotted in a color scale at the bottom (using a filled contour plot). The black curves correspond to the center of spinal MN activity (CoA). Right panel: mean (+SD across subjects, and each point represents the data for an individual child) correlation coefficient between CoAs of individual subjects with the averaged CoA during NW (shown on the left panel). Asterisk denotes significant difference across conditions (RM ANOVA with Tukey's HSD, *p* < 0.05). **(B)** Temporal characteristics of lumbar and sacral segmental activation. Left panels: polar plots of the maximum activity timing of sacral (S1 + S2) and lumbar (L3 + L4) segments for each condition (same format as in Figure 2B). Asterisk denotes significant difference for the NW condition (circular Watson-Williams test *p* < 0.05). Right panel: similarity (correlation) of spinal segmental activation across the two walking conditions. Note poor if any correlation between Exo and NW.

Mapping the EMG-activity profiles onto the rostrocaudal anatomical location of MN-pools in the lumbosacral spinal cord highlighted specific variations in the spinal maps of α-MN activity during Exo walking. Figure 3A (left panels) shows the averaged segmental motoneuron output over the step cycle. To quantify the similarity of those spinal maps, we computed the correlation of individual subject's CoA with averaged CoA during NW and we found a significant difference in the Exo condition (*p* < 0.01, One-way ANOVA). During NW, these correlations were relatively high (*r* = 0.74 ± 0.19) and the CoA resembled that of adults during level walking (Ivanenko et al., 2006), while during Exo walking the correlations were lower and more variable across children (*r* = −0.24 ± 0.69) (Figure 3A, right panel).

The prominent feature of these maps in NW was a distinct activation of lumbar and sacral segments during early and late stance, respectively. The output pattern of each segment is shown in the top panels of Figure 3A and one can recognize the major loci of lumbar and sacral activity around touchdown and push-off, associated with the activity of proximal and distal extensors correspondingly. We quantified it by computing the maximum activity timing of the upper lumbar (L3 + L4) and sacral (S1 + S2) output (Zhvansky et al., 2022). The NW condition showed distinct activation of the lumbar and sacral segments during early and late stance (*p* < 0.05, Watson-Williams test), whereas the Exo condition showed no differentiation (*p* > 0.05, Figure 3B, left panels) and a tendency for synchronous activation of the lumbar and sacral segments. Furthermore, the activation of the lumbar and sacral segments during Exo walking showed no or only minor similarities with NW (Figure 3B, right panel).

**Figure 4:**
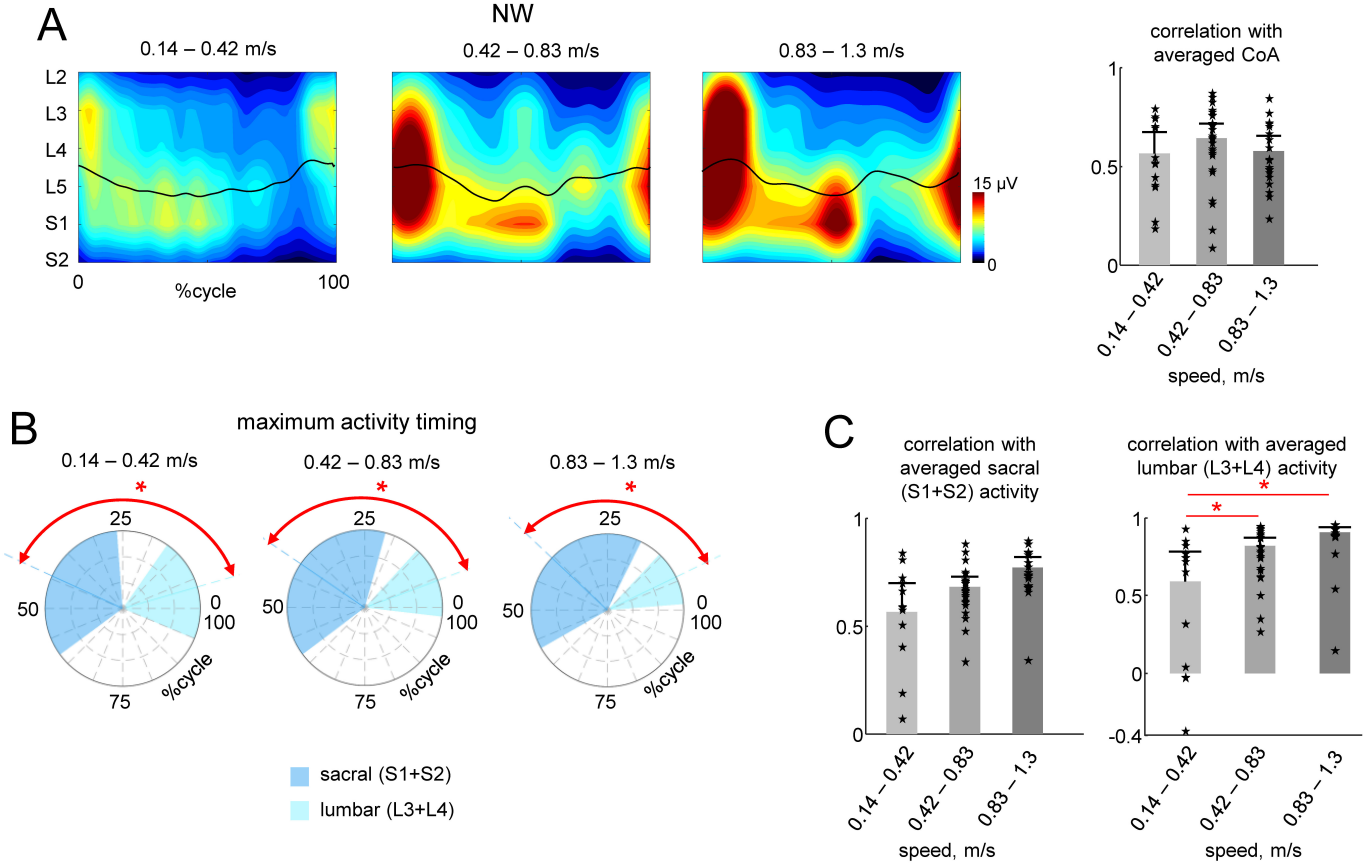
Effect of speed during NW. **(A)** Spatiotemporal maps of motoneuron activity of the lumbosacral enlargement in three different ranges of walking speed (same format as in Figure 3A). Spinal maps were reconstructed using ensample-averaged EMG envelopes of all strides within each diapason of walking speeds. **(B)** Polar plots of the maximum activity timing of sacral (S1 + S2) and lumbar (L3 + L4) segments (as in Figure 3B). **(C)** Correlations (+SD across strides) of individual strides' CoA with averaged CoA during NW (as in Figure 3A) and similarity (correlation) of lumbar and sacral segmental output with the averaged output during NW (as in Figure 3B). We also plotted the individual data for all strides analyzed [13 strides from two subjects for the lower walking speeds (0.14–0.42 m/s), 24 strides from all six subjects for the middle speed (0.42–0.83 m/s) and 19 strides from four subjects for faster speeds (0.83–1.3 m/s)]. Asterisks denote significant differences (RM ANOVA with Tukey's HSD, *p* < 0.05) across walking speed ranges. Note generally high (0.6–0.9) correlations illustrated in panels **(B, C)** for all speed ranges, in contrast to the lack of correlations between NW and Exo conditions (sf. Figure 3 right panels).

Given the speed difference between the two conditions (Exo and NW), to assess the potential influence of speed, we separated the strides during NW into three speed ranges: low (0.14–0.42 m/s), medium (0.42–0.83 m/s), and high (0.83–1.3 m/s). We performed a similar analysis to that in Figure 3, comparing the spinal maps features across the strides. Figure 4A shows the averaged spinal maps during NW for each speed range. In contrast to Exo walking (Figure 3), the correlation of CoAs with the averaged CoA was relatively higher (>0.6) and did not depend on speed (Figure 4A, right panel). The maximum activity timing for lumbar and sacral output was differentiated for all speed ranges (*p* < 0.01, Watson-Williams test, Figure 4B), and the correlation of lumbar and sacral activity with the averaged (across all speeds and participants) output was higher than 0.6 for all speed ranges (Figure 4C).

## Discussion

The aim of the present study was to investigate the spinal motor output characteristics during walking in children with the ExoAtlet Bambini exoskeleton that assists leg movements and locomotor training. The results on variations in muscle activity patterns and motor pool activation (Figures 2, 3) add to our knowledge of human-machine interactions and exoskeleton technology; they also highlight the necessity of activating spinal neural networks in order to approximate the functional neuromechanics of the spinal locomotor circuitry.

### Beneficial effect of exoskeleton-assisted gait in children on muscle activity

We investigated the effect of walking in the exoskeleton on the muscular activation patterns in typically developing children. During development, gait characteristics gradually evolve toward mature gait (Forssberg, 1985; Yang et al., 2015; Payne and Isaacs, 2017; Miyagishima et al., 2023). For instance, at the onset of independent walking the pendulum mechanism of walking is lacking and each child needs to learn it (Ivanenko et al., 2007). At an early age, there is a notable amount of muscle coactivation and variability (Teulier et al., 2012; Sylos-Labini et al., 2022; Hinnekens et al., 2023), whereas fine tuning and maturation of muscle activity patterns continues throughout childhood (Ivanenko et al., 2013; Cappellini et al., 2016). We recorded walking in participants aged 7–11 years old to evaluate performance in children of similar ages and to rule out the potentially confusing effect of age. Robotic devices are being widely employed for improving gait in children with developmental disorders and, in particular, we examined and implemented here the method for assessing the neuromechanics of spinal locomotor output during exoskeleton-assisted gait.

While children were instructed not to attempt to walk voluntarily and to remain passive, adaptation to the moving exoskeleton was expected. For instance, even when children were standing in the exoskeleton we noticed postural activity in the calf muscles (although we did not record it). Nevertheless, the fact that children could not fully relax in the exoskeleton is interesting *per se*. It is also worth noting that passive rhythmic movements may activate the central pattern generators in the spinal cord (Rossignol et al., 2006; Selionov et al., 2009), and full foot loading and postural activity in the exoskeleton may further contribute to the observed leg muscle activation (Figure 2). Overall, during normal and exoskeleton-assisted walking, children displayed prominent lower limb muscle activation, including activity related to limb loading during stance and activity for the swing phase (Figure 2). The involvement of muscles during assisted walking in the exoskeleton is an interesting finding that supports some earlier research (e.g., Sylos-Labini et al., 2014). Surprisingly, despite slower speed and assistance in both posture and leg movements, overall muscle activity in children was not reduced, as one would expect. This can likely be accounted for by the important contribution of afferent feedback to the pre-programmed motoneuronal drive (Nielsen and Sinkjaer, 2002), and the “active” nature of stepping in the exoskeleton (the child was not fully “relaxed”, experienced full foot loading during stance and helped to maintain the upper trunk posture) even though the limb movements were guided by the exoskeleton. Because the overground exoskeleton allows for full body-weight foot loading during stance and full unloading during swing (Shapkova et al., 2020), and because the hips and knees are relatively well-matched (Figure 1), the exoskeleton serves as a major source of sensory signals during walking. For instance, the signals coming from afferents activated by hip extension and from various load receptors regulate the transition from stance to swing, reinforce force feedback and promote phase-dependent modulation of muscle activity (Duysens et al., 2000; Pearson, 2004). It would be interesting to investigate the role that sensory inputs play in the observed deviations in motor pool activity (Figure 3), however, the interpretation of muscle forces and sensory contributions is rather challenging and requires a dynamic model of the human exoskeleton system and neuromechanical simulations with integrated sensory commands (Di Russo et al., 2023).

The beneficial effect of the exoskeleton for engaging muscle activity patterns (Figure 2) is a notable finding from this study, albeit variances in muscle activity patterns should be noted with respect to NW. Variations in muscle activity have also been documented in other orthosis-assisted or unloaded walking conditions recorded in adults (Hidler and Wall, 2005; Van Asseldonk et al., 2008; Moreno et al., 2013; Ramanujam et al., 2018). For example, with body weight unloading, the distal leg extensors decrease their activity, whereas the proximal extensors (quadriceps) show a “paradoxical” increase in activation and hamstring muscles may demonstrate significant changes in the activation waveforms (Ivanenko et al., 2002). In this study, we also observed a tendency for increasing quadriceps activity (Figure 2, right panels), as well as changes in its pattern and center of activity (Figure 2B). The results seem to corroborate the previous findings in adults on walking in the powered exoskeleton showing that the most varied EMG activity is observed in the proximal muscles: quadriceps in children in the current investigation (Figure 2B) and in adults in Ramanujam et al. (2018), and hamstring in Sylos-Labini et al. (2014) and Swank et al. (2019). In addition, the distal extensors lacked a prominent activity burst at the end of stance during Exo walking (the CoA shifted toward midstance, Figure 2B) related normally to push-off and anticipatory control of the center-of-mass vertical oscillations during step-to-step transitions that gradually develops in adolescence toward mature gait (Nuñez-Lisboa et al., 2023). The exoskeleton's limited range of motion in the ankle joint may contribute to this missing feature of distal extensor activation during step-to-step transitions, although it may also be related to the fact that the exoskeleton aids in full control of whole-body motion and does not necessitate adherence to the specifics of the pendulum mechanism walking control of the center-of-mass (see the following section).

### Spinal locomotor output during walking in the exoskeleton and clinical implications

Unlike techniques that look for individual muscle activities or shared regularities in muscle activation of particular muscle groups, the spinal maps approach (Figure 3) is based on an evolutionary adapted MN grouping of distinct muscles in the spinal cord and the corresponding motor pool activation (Yakovenko et al., 2002; Ivanenko et al., 2013). The analysis of the spinal maps highlighted differential changes in the activation of sacral and lumbar segments during NW and its lacking during Exo walking (Figure 3). It is unlikely that coactivation of lumbar and sacral segments (Figure 3A) was attributed to slower walking in the exoskeleton. For instance, the EMG amplitudes during NW at slow speeds are indeed generally significantly smaller and some activations may be missed (Ivanenko et al., 2006). However, walking in the Exo did not result in vanishing of muscular activity, and the activity even increased in some muscles (Figure 2A), so that we compared Exo and NW at comparable total spinal MN activity level. Furthermore, even though we did not record Exo walking at higher speeds, we evaluated the effect of speed on the spatiotemporal characteristics of the motor pool output during NW (Figure 4). Despite some variability, the similarity of the CoA and the lumbar and sacral segmental output was generally higher in the three walking speed ranges during NW (Figure 4). In sum, while the exoskeleton gait provided basic patterns of muscle activation (Figure 2), it missed some aspects associated with the establishment of typical spatiotemporal spinal motor pool activity, e.g., demonstrating an aberrant activation of lumbar segments (Figure 3).

What is the importance of the above-mentioned variations in muscle activity and spinal maps for the neuromechanics of the pattern generation circuitry and gait control? A thorough understanding of the functional spinal neural circuits can help design focused therapy approaches and promote the advancement of neuroprosthetics that help patients with locomotor impairments regain their functional movement patterns. The naturalness of gait patterns for walking in an exoskeleton has been addressed in a number of studies (Sylos-Labini et al., 2014; Ramanujam et al., 2018; Swank et al., 2019) and it has also been explored for the benchmarking of exoskeleton assessments (Remazeilles et al., 2022; Zhvansky et al., 2022; Rodrigues-Carvalho et al., 2023). When evaluating the naturalness of exoskeleton-assisted gait in patients with locomotor deficits, one reason to use spinal maps of motor pool activation is to compare the neural control strategy to normal walking, since an abnormal spatiotemporal integration of spinal motor activity carries a risk of failure or abnormalities in gait recovery (Avaltroni et al., 2024). Consideration of the spinal maps of motor pool activity is further supported by the promising approach of neuromodulating the spinal circuitry. For instance, it has been demonstrated that successful rehabilitation of spinal cord injured patients results from using the location and timing of epidural spinal cord electrical stimulation tailored on the specific features of spinal cord topology so as to reproduce the natural spatiotemporal activation of motor pools (Rowald et al., 2022). Thus, activating and modifying the spinal neural pathways in order to approach the normal evolutionary evolved neuromechanics of the functioning of the spinal locomotor circuitry for bipedal gait is a common objective of such studies.

There are various approaches for optimizing exoskeleton controllers for gait rehabilitation (Baud et al., 2021) that include recent initiatives for benchmarking the exoskeleton performance (Remazeilles et al., 2022; Zhvansky et al., 2022; Rodrigues-Carvalho et al., 2023). While predefined gait trajectory control is the most common assistive control strategy for exoskeletons, minimizing or adapting exoskeleton torques may also be helpful and effective. For instance, the unique feature of the “Angel Legs M20” exoskeleton for assisted gait in children (Choi et al., 2024) is that the torque at the hip and knee joints can be adjusted according to the patient's residual muscle strength, allowing more active participation, variability of movements, and dynamic gait pattern adaptation. Although, we did not record the force/torque profiles, they can vary depending on the behavior of the child (and the accompanying person to some extent) and likely require a precise dynamic model of the human exoskeleton system for their interpretation. For instance, we have previously recorded torque profiles during exoskeleton walking in both healthy individuals and spinal cord injury patients (Sylos-Labini et al., 2014) and it is difficult to unambiguously associate them with the EMG patterns of individual muscles. Therefore, the spinal maps of MN activity may further assess the naturalness of gait patterns induced by such training (a global view of ensemble alpha motoneurons activity) or for optimizing the exoskeleton controllers by aligning with the natural walking pattern of the person.

It is noteworthy that the spinal maps of MN activation can provide indirect insights into the activity of premotor interneurons, which constitute the core of central pattern generators of locomotion (CPG) (Kiehn, 2016; Grillner and El Manira, 2020). Whether or not MNs are an integral part of CPGs, they play a role in determining the rhythm and patterns of locomotion by feeding information back to the upstream circuitry (Barkan and Zornik, 2019). The improper activation of motor pools can give information regarding abnormal spatiotemporal integration of activity in specific spinal segments and may serve as a marker of certain disorders (Avaltroni et al., 2024). In order to generate functional plasticity of spinal centers and simulate the normal functional neuromechanics of the spinal locomotor circuitry, this may be particularly important for long-term adaptation to exoskeleton training and for gait rehabilitation.

In addition, one cannot exclude important individual differences in exoskeleton stepping adaptation. Thus, we observed a notable amount of inter- and intra-individual variability (see SDs in Figures 1, 2 and Table 1). While the ExoAtlet Bambini exoskeleton follows the predetermined pattern of leg movement, the reported kinematic variability (Figure 1; Table 1) can be attributed to differences in the children anthropometric data and to overground stepping with some influence of weight transfer between the legs and dynamic interactions. For instance, stride-to-stride variability of stride duration and inter-joint coordination was also previously reported to be higher during exoskeleton overground walking compared to treadmill walking (van Hedel et al., 2021). Concerning stride-to-stride and inter-subject variability in the EMG patterns, it is also common for normal walking especially at slow speeds and in proximal leg muscles (Winter and Yack, 1987; Sylos-Labini et al., 2017). On the other hand, while stride-to-stride variability can made it difficult to ascertain the effects of exoskeleton assistance (Bulea et al., 2018), it is beneficial for gait rehabilitation. Effective neurorehabilitation requires active engagement. Children that receive overground gait training are able to explore and navigate in a more natural setting, accomplish greater stride-to-stride variability, achieve more effective dynamic balance control and proper body alignment during weight-shifting actions, and adapt their neuromuscular system. Whatever the exact reasons for the reported inter-individual differences in adaptation to the exoskeleton gait (Figure 2B), we also observed some systematic features in the motor pool activation such as a weaker differentiation of sacral and lumbar activity in the Exo condition in all children (Figure 3B).

Spinal maps may provide information on certain differences in the global characteristics of the spinal locomotor controllers that could be compromised when wearing an exoskeleton while walking. During normal walking, the center of body mass (COM) vaults over the stance leg, resulting in the exchange of the COM's potential and kinetic energy, and this behavior is inherently linked to the optimization of movements in children (Cavagna et al., 1983; Saibene and Minetti, 2003; Ivanenko et al., 2007). The differential involvement of the lumbar and sacral motor pools during weight acceptance (onset of stance) and propulsion (end of stance) and the accompanying CoA oscillations are essentially related to the control of the COM (Cappellini et al., 2010; Dewolf et al., 2019). The results point to the lack of characteristic lumbosacral oscillations of motor pool activity during exoskeleton-assisted gait in children (Figure 3). In addition, as previously mentioned, the distal extensors lacked a prominent activity burst at the end of stance (Figure 2B), which is normally associated with anticipatory control of the COM vertical movements during step-to-step transitions (Nuñez-Lisboa et al., 2023). Possibly, because the exoskeleton aids in full control of limb movements and whole-body motion, there is no need to adhere to the specific features of the pendulum mechanism control of walking, which is thought to be related to the energetics of bipedal gait (Alexander, 1989). For instance, walking in the exoskeleton does not seem to decrease the total level of muscle activity in healthy children (Figure 2A) and the total level of MN activity is even notably higher when comparing similar ranges of walking speed in NW (cf. Figures 3A, 4A). Alternatively, it could be related to some changes in the biomechanical requirements when walking in the exoskeleton. For example, the exoskeleton may be acting as disturbances to the subject due to the intermittent contacts between the exoskeleton and the subject, or a lack of prominent extensor activity at the end of stance (Figure 2) could be attributable to restricted changes in the ankle joint angle at push-off (Figure 1). Whatever the exact reasons for the lack of typical spatiotemporal motor pool activity (Figure 3), it should be taken into account when applying exoskeletons for gait rehabilitation or for exoskeleton performance assessments in order to assist children developing their natural gait generation pattern.

## Limitations

This study had several limitations. First, we tested it on a small sample of subjects, but even for children in a limited age range (7–11 yrs), the results indicate distinct alterations in the motor pool activation (Figures 3, 4) and also suggest additional research into an intriguing age-related effect, considering that the spinal locomotor output gradually changes with age (Ivanenko et al., 2013). Second, for children, walking in an exoskeleton is a novel task that could take some getting used to in order for them to adapt to it more optimally, while in this study we simply permitted a brief amount of training before gait recording. This issue is especially important for long term adaptations during gait rehabilitation and for children with neurological disorders since a certain threshold for repetition of the same task, at least hundreds of repetitions, must be crossed to induce effective experience-dependent neuroplasticity of pattern generation network and brain reorganization to achieve permanent change at the synaptic level (Kleim and Jones, 2008). Third, we recorded a limited number of muscles (in part due to limitations in placing EMG electrodes on the lower limb strapped to the exoskeleton, Figure 1A), but we have previously shown that the major loci of MN activity are similar and can be determined by using a set of recorded muscles representing major extensor and flexor groups innervated from the lumbosacral enlargement (Ivanenko et al., 2013; La Scaleia et al., 2014). Finally, lower limb movements were assisted only in the sagittal plane, at a relatively slow speed and in a certain regime of joint movement controllers (e.g., with limited ankle joint angle motion). Given a heterogeneity of existing pediatric robotic devices for gait training, some of the suggestions in this article based on the analysis of the spinal locomotor output may be generalized or revised as empirical data on the human–exoskeleton interactions will be accumulated according to the robot device type and also to the patient profile.

## Conclusions

Exoskeleton technology holds significant potential to improve locomotion in children with gait impairments and to enhance autonomy and involvement in daily activities. Overall, the results support the notion that exoskeleton gait can offer basic patterns of muscle activation (Figure 2), although it may miss certain features linked to the formation of typical spatiotemporal motor pool activity (Figure 3), normally associated with the control of the center of body mass motion and the pendulum mechanism of bipedal walking. Future research can also explore the learning and adaptation effects of exoskeleton-assisted gait during long usage. This approach may prove to be a valuable method for directing the optimisation of robotic systems to support children in the development of their natural gait pattern.

## Data Availability

Data will be made available upon reasonable request.
